# Pyrenoid functions revealed by proteomics in *Chlamydomonas reinhardtii*

**DOI:** 10.1371/journal.pone.0185039

**Published:** 2018-02-26

**Authors:** Yu Zhan, Christophe H. Marchand, Alexandre Maes, Adeline Mauries, Yi Sun, James S. Dhaliwal, James Uniacke, Simon Arragain, Heng Jiang, Nicholas D. Gold, Vincent J. J. Martin, Stéphane D. Lemaire, William Zerges

**Affiliations:** 1 Department of Biology & Centre for Structural and Functional Genomics, Concordia University, Montreal, Quebec, Canada; 2 Laboratoire de Biologie Moléculaire et Cellulaire des Eucaryotes, Institut de Biologie Physico-Chimique, UMR8226, CNRS, Sorbonne Universités, UPMC Univ Paris 06, 13 rue Pierre et Marie Curie, Paris, France; Youngstown State University, UNITED STATES

## Abstract

Organelles are intracellular compartments which are themselves compartmentalized. Biogenic and metabolic processes are localized to specialized domains or microcompartments to enhance their efficiency and suppress deleterious side reactions. An example of intra-organellar compartmentalization is the pyrenoid in the chloroplasts of algae and hornworts. This microcompartment enhances the photosynthetic CO_2_-fixing activity of the Calvin-Benson cycle enzyme Rubisco, suppresses an energetically wasteful oxygenase activity of Rubisco, and mitigates limiting CO_2_ availability in aquatic environments. Hence, the pyrenoid is functionally analogous to the carboxysomes in cyanobacteria. However, a comprehensive analysis of pyrenoid functions based on its protein composition is lacking. Here we report a proteomic characterization of the pyrenoid in the green alga *Chlamydomonas reinhardtii*. Pyrenoid-enriched fractions were analyzed by quantitative mass spectrometry. Contaminant proteins were identified by parallel analyses of pyrenoid-deficient mutants. This pyrenoid proteome contains 190 proteins, many of which function in processes that are known or proposed to occur in pyrenoids: *e*.*g*. the carbon concentrating mechanism, starch metabolism or RNA metabolism and translation. Using radioisotope pulse labeling experiments, we show that pyrenoid-associated ribosomes could be engaged in the localized synthesis of the large subunit of Rubisco. New pyrenoid functions are supported by proteins in tetrapyrrole and chlorophyll synthesis, carotenoid metabolism or amino acid metabolism. Hence, our results support the long-standing hypothesis that the pyrenoid is a hub for metabolism. The 81 proteins of unknown function reveal candidates for new participants in these processes. Our results provide biochemical evidence of pyrenoid functions and a resource for future research on pyrenoids and their use to enhance agricultural plant productivity. Data are available via ProteomeXchange with identifier PXD004509.

## Introduction

Photosynthesis occurs in organelles called chloroplasts, in both plants and algae. Chloroplasts themselves contain specialized compartments. The light-driven reactions of photosynthesis and ATP synthesis occur in membranous vesicles called thylakoids. Certain metabolic pathways occur in the chloroplast stroma, a proteinaceous aqueous compartment which is enclosed by the chloroplast envelope. Lipid metabolism and storage occur in plastoglobuli. Functions of each of these compartments have been elucidated by proteomics; *i*.*e*. the use of mass spectrometry and bioinformatics to carry out large scale, comprehensive characterizations of the protein composition or “proteome” of a purified intracellular compartment [1–4].

The pyrenoid is a microcompartment within the chloroplasts of algae and hornworts. Its known function is to promote photosynthetic CO_2_ fixation by the enzyme ribulose-1,5-bisphosphate carboxylase/oxygenase (Rubisco). It does so by mitigating the low catalytic efficiency of Rubisco which results from a dual substrate specificity of the active site for CO_2_ or O_2_ [5]. CO_2_ is the substrate for the productive carbon-fixing step of the Calvin-Benson cycle in photosynthesis. An oxygenase activity of Rubisco leads to the production of glycolate to be recycled by the non-productive and energetically-costly photorespiratory pathway [6]. The pyrenoid also mitigates the limited availability of CO_2_ in aquatic environments where, compared to the atmospheric conditions of land plants, uptake is hampered by slower diffusion rates and the conversion of most CO_2_ to the bicarbonate anion, which cannot diffuse across the lipid bilayers of the plasma membrane and chloroplast envelope membranes [7]. The CO_2_ concentration is enhanced around the major pool of Rubisco localized in the pyrenoid by a “carbon concentrating mechanism” (CCM). This system imports bicarbonate using membrane transporters and converts it to CO_2_ within the pyrenoid using carbonic anhydrases. Bicarbonate diffuses within membranous tubules which are contiguous with thylakoid vesicles and extend into the pyrenoid [7, 8]. Within these pyrenoid tubules are “mini-tubules” which might serve as a conduit for diffusion of bicarbonate and metabolites between the pyrenoid interior or “matrix” and the stroma [9]. In addition, the pyrenoid is believed to sequester Rubisco from O_2_ produced by photosystem II (PSII) complexes in thylakoid membranes located throughout the rest of the chloroplast [10].The pyrenoid is functionally analogous to the carboxysomes of cyanobacteria, in that both microcompartments provide Rubisco with a low O_2_/CO_2_ ratio to favor its carboxylase activity [11].

Other functions of pyrenoids have been proposed, for example, in the synthesis of the starch that surrounds them in a dense sheath [12] and in the maintenance and expression of the genome of the chloroplast [13–16]. However, our understanding of pyrenoid functions is incomplete because it is largely based on evidence from microscopy.

Biochemical and proteomic analyses of pyrenoids have been hampered by their instability during cell breakage and fractionation [10]. In a previous study, pyrenoid-enriched fractions of the green alga *Chlamydomonas reinhardtii* were obtained by stabilizing the pyrenoids in cell lysates with mercury chloride [17]. These pyrenoid-enriched fractions were shown to contain both subunits of Rubisco and a starch synthase. Other proteins were detected, but not identified. Mackinder et al. recently reported results of proteomic analysis which identified Chlamydomonas proteins present in a subcellular pyrenoid-containing fraction and showing a direct correlation of their abundance with conditions that favor pyrenoid growth (low [CO_2_]) [18]. In contrast to the present study, they did not report a functional characterization of the pyrenoid but focused on the characterization of a major pyrenoid protein, EPYC1, revealing it has a structural role in linking Rubisco holoenzyme complexes within the pyrenoid [18].

Here, we use a proteomic approach to reveal functions of the pyrenoid in *C*. *reinhardtii*. We improved published methods for the isolation of pyrenoids and chloroplasts [19, 20]. Pyrenoid-enriched fractions were subjected to a quantitative proteomic analysis using accurate mass/high resolution tandem mass spectrometry (ESI-MS/MS). To identify protein contaminants for subtraction, we analyzed fractions obtained from pyrenoid-deficient mutants. The resulting pyrenoid proteome contains 190 proteins. Functional annotations of the proteins were used to interpret pyrenoid functions. This proteome provides directions for the exploration of pyrenoid functions and establishes an approach for more comprehensive characterizations of pyrenoid functions, for example, under changing biotic and abiotic conditions.

## Materials and methods

### Strains and culture conditions

All strains were cultured in Tris-Acetate-Phosphate medium [21] at 25°C, with orbital shaking, to a density of 2–4 x10^6^ cells/ml under dim indirect light (approximately 8 μmoles/m^2^/s). *KA6* is wild-type for photosynthesis and has a severe cell-wall defect (*CW15*) which can be seen as rapid (within 20 sec) cell lysis in 2.0% (v/v) Triton X-100. *ΔrbcL* carries the disrupted *rbcL* gene of CC-4696 (from the Chlamydomonas Resource Centre and Drs. Robert Spreitzer and Genhai Zhu, University of Nebraska) which was crossed into a *CW15* genetic background in order to weaken its cell wall for lysis in Triton X-100, as part of the pyreniod enrichment protocol described in the next subsection. *ΔrbcL* lacks the large subunit of Rubisco and, consequently, also lacks the Calvin-Benson cycle and photorespiration. *SSAT* was generated previously by complementation of the *ΔRBCS* mutant by one of the genes encoding the small subunit of Rubisco from *Arabidopsis thaliana*, *RBCS1B*. *SSAT* assembles a hybrid Rubisco holoenzyme that is expressed at wild-type levels and appears to have near normal rates of carboxylation [22]. Nonetheless, *SSAT* displays reduced levels of growth under photoautotrophic conditions in air, likely because it lacks the known carbon concentrating activity of pyrenoid [23]. *SSAT* is, nevertheless, photosynthetic (photoautotrophic) and, therefore, its metabolism is likely close to that of the pyrenoid-containing strain *KA6*.

### Generation of pyrenoid-enriched fractions

**Fig 1:**
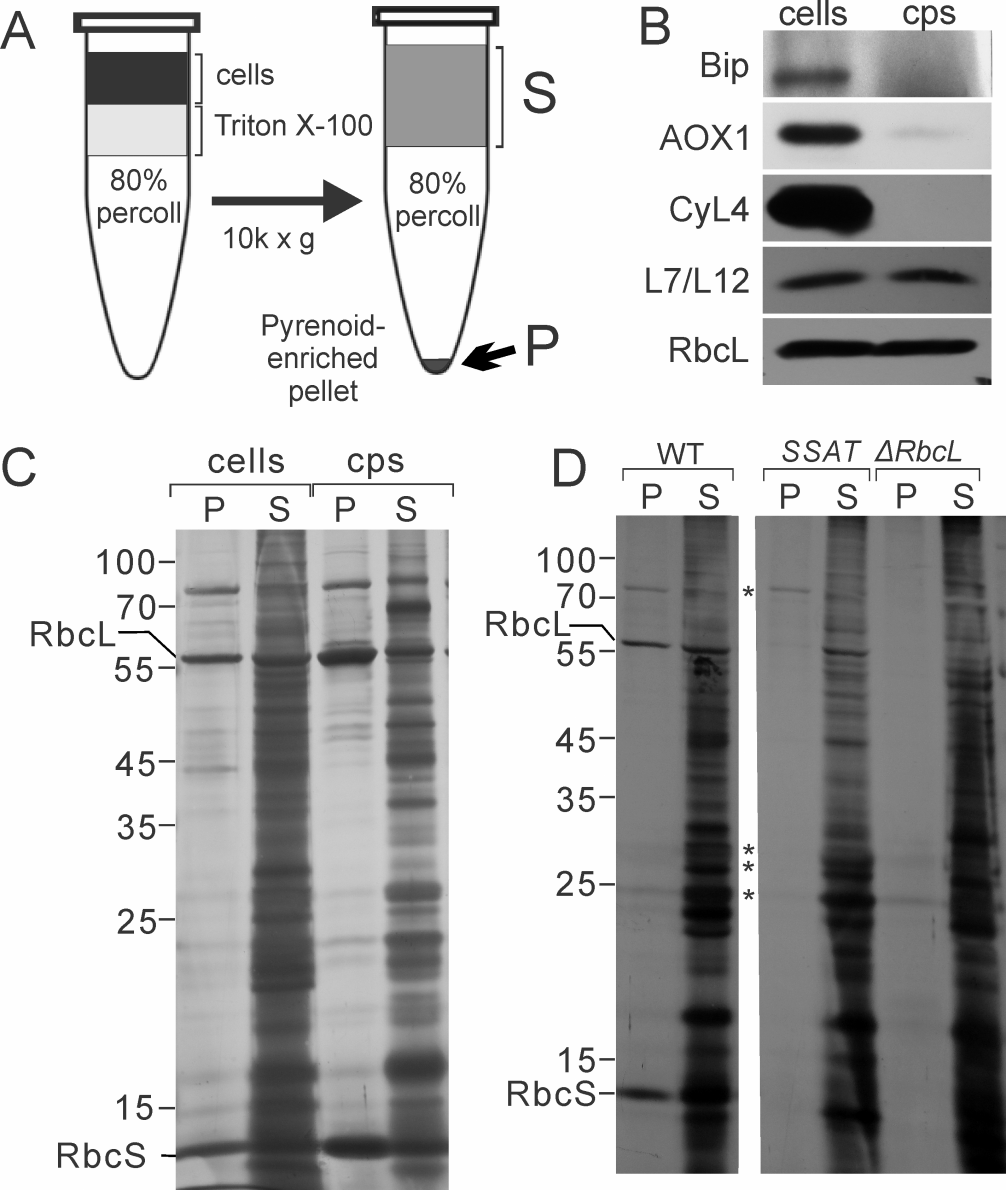
Purification and analysis of pyrenoid preparations. (A) Pyrenoid-enriched pellet (P) fractions were obtained by solubilizing cells or purified chloroplasts (cps) with Triton X-100 followed by immediate isolation of pyrenoids by centrifugation through a Percoll cushion. Detergent-solubilized material remained in the supernatant (S). Pyrenoids and other material were recovered in the pellet (P). (B) Purification of the chloroplasts from which pyrenoid–enriched fractions were prepared is demonstrated by results of immunoblot analyses comparing extracts of cells and chloroplasts (cps) for the relative levels of marker proteins for ER (Bip), mitochondria (AOX1), cytoplasm (CyL4), and the chloroplast (L7/L12 and RbcL). Samples with 1.0 μg chlorophyll were loaded in each lane. (C and D) Results of SDS-PAGE and silver-staining reveal proteins of the P and S fractions from (C) cells and isolated chloroplasts and (D) WT and the pyrenoid-deficient control strains SSAT and *ΔrbcL*. (*D*) Asterisks indicate bands that appear to be contaminants common to P fractions from WT and at least one pyrenoid-deficient mutant. The P and S represent proportional loading of protein isolated from material containing 65 μg chlorophyll.

Strains used for the isolation of chloroplasts and pyrenoids must have a weakened cell wall. This was achieved by using strains with the CW15 genetic background; a polygenic weakened cell wall phenotype. The CW15 phenotype eliminated the requirement for chemical fixation to stabilize pyrenoids (e.g. with mercury chloride [26, 27]) because rapid lysis in Triton X-100 allows the purification of the pyrenoids, but not solubilized material, by centrifugation through a Percoll cushion. Cells were pelleted by centrifugation at 5,000 x g at 4°C. The pellet was resuspended in Breaking Buffer (BB) (300 mM sorbitol, 10 mM Tricine pH 7.8, 5 mM EDTA) to a density of 4 x 10^7^ cells/ml. A 1.0 ml aliquot of 80% (v/v) Percoll (GE Healthcare) in BB (prepared by mixing 800 μl Percoll with 200 μl of 5X BB stock; 1.5 M sorbitol, 50 mM Tricine pH 7.8, 25 mM EDTA) was dispensed into a 1.5 ml microcentrifuge tube. This Percoll cushion was gently overlaid with 250 μl of freshly prepared 2.0% (v/v) Triton X-100 (Sigma-Aldrich) in BB, avoiding mixing of the phases. Cells or isolated chloroplasts (250 μl in BB) were then pipetted into the upper phase of Triton X-100 (mixing with the 80% Percoll phase was avoided) and the tube was immediately centrifuged at 10,000 x g for 10 min at 4°C. The uppermost 500 μl of the supernatant was removed and designated the S fraction (Fig 1A). The remaining supernatant was discarded. The pellet was rinsed once by gently adding and removing 200 μl of ice cold BB without resuspension. The supernatant (S) and pellet (P) fractions can be stored at -80°C. For the pyrenoid isolation from chloroplasts, the chloroplasts were first isolated as described previously [19] with the following modifications. Cells were incubated in 0.5% saponin (wt/vol) (Sigma-Aldrich) in BB for 15 min before this suspension was passed through the syringe. Steps of pyrenoid purification from isolated chloroplasts were as described above for intact cells except that the Triton X-100 concentration was 0.5% (vol/vol). The effectiveness of chloroplast isolation is demonstrated by immunoblot analyses in Fig 1B; marker proteins for the chloroplast (RbcL and the L7/L12 protein of the chloroplast ribosome) were retained while proteins for endoplasmic reticulum (Bip), mitochondria (AOX1), and cytoplasm (CyL4) were severely depleted.

### Immunofluorescence staining

Freshly isolated pyrenoids, obtained from WT cells and in 100 μl BB, were fixed with 4.0% (w/v) paraformaldehyde (BioShop) for 10 min at room temperature. The pyrenoids were then collected by centrifugation at 10,000 x g for 5 min. The pellet was washed twice with BB, resuspended in 100 μl BB, and then immuno-reacted with affinity-purified antibodies against RbcL or RbcS (1:1,000 dilutions, Dr. Spreitzer, University of Nebraska) for 75 min at room temperature with gentle agitation. The conjugates were washed twice with BB, by centrifugation (5,000 x g for 10 min) and resuspension in BB, and finally resuspended in 100 μl BB. This pyrenoid suspension was incubated with Alexa Fluor 488 AffiniPure Goat Anti-Rabbit IgG secondary antibody (1:200, Invitrogen) for 45 min at room temperature with gentle agitation. The pyrenoid-antibody conjugates were washed again in BB and resuspended in 50 μl BB. Samples were processed as previously described [28] and observed with a Leica DMI 6000 microscope (Leica) with a 63X/1.4 objective, a Hamamatsu OrcaR2 camera, and Volocity acquisition software (Perkin-Elmer) in the DIC and GFP channel.

### Protein electrophoresis, silver staining

Proteins were extracted as described previously [29]. Proteins were resolved by 12% SDS-PAGE [30] and visualized by silver-staining according to the manufacturer’s protocol (Sigma-Aldrich).

### Immunoblot analyses

Proteins in the gel were transferred to a polyvinylidene difluoride membrane (BioRad) [30]. The membrane was blocked using 5.0% (wt/vol) dried non-fat milk in Phosphate Buffer Saline (137 mM NaCl, 2.7 mM KCl, 10 mM Na_2_HPO_4_, 1.8 mM KH_2_PO_4_) supplemented with 0.1% (v/v) Tween-20) and incubated in the presence of primary antibody for 2 h at room temperature. Primary antibodies were directed against the mitochondrial protein AOX1 (1:20,000 dilution; Agrisera), the ER protein BiP (1:200 dilution, Agrisera), the cytoplasmic ribosomal protein CyL4 (1:10,000 dilution) [31], the chloroplast ribosomal protein L7/L12 (1:4000 dilution) [32] and RbcL (1:4,000 dilution, Dr. Robert Spreitzer, University of Nebraska). Proteins of interest were detected by enhanced chemiluminescence (Pierce) using a horseradish peroxidase-conjugated anti-rabbit secondary antibody (1:10,000 dilution, Sigma Aldrich) and X-ray film.

### *In vivo* ^35^S-pulse-labeling

^35^S-pulse-labeling was performed for 5 min with 1.2 x 10^7^ cells in 0.3 ml of TAP medium lacking NH_4_SO_4_. Cycloheximide was added to a final concentration of 10 μg/ml 5 min prior to the addition of 80 μCi of [^35^S]SO_4_. Cells were not deprived of SO_4_ for more than the ~15 min required for centrifugation and resuspension and treatment with cycloheximide prior to pulse-labeling. Pulse-labeling was carried out on an orbital shaker for 5 min under white light intensity of approximately 80 μmoles/m^2^/s. Cells were pelleted by centrifugation at 4,000 x g for 2 min, resuspended in BB. P and S fractions were prepared from these cells as described above (Fig 1A). P and S fractions were complemented up to 100 μl SDS-PAGE loading buffer (250 mM Tris pH 6.8, 2% (wt/vol) SDS, 20% (vol/vol) glycerol, 50 mM DTT, 2% (vol/vol) 2-mercaptoethanol), incubated at RT for 60 min, and then 50 μl of each sample was loaded on a 13% denaturing SDS-polyacrylamide gel (with 8M urea). Following electrophoresis, the gels were silver stained, photographed and then dried. ^35^S-labelled proteins were revealed by phosphorimaging.

### Mass spectrometry

Proteomics grade endoproteinases (Lys-C and Trypsin Gold) and ProteaseMax surfactant were purchased from Promega (Charbonnières, France). Reversed phase C18 spin columns, precolumns and analytical columns were all obtained from Thermo Scientific (Les Ulis, France). Solvents and ion-pairing agents were certified LC-MS grade and all other chemicals were purchased from Sigma-Aldrich (Saint-Quentin Fallavier, France) with the highest purity available. Pyrenoid pellets were washed with prechilled acetone and maintained at -20°C for at least 2 hours. After centrifugation (21,500 x g, 10 min, 4°C), pellets were dissolved in 60 μL of 50 mM ammonium bicarbonate containing 6.5 M urea, 5 mM dithiothreitol (DTT) and 0.05% (v/v) ProteaseMAX surfactant at 30°C for 30 min. Free cysteines were alkylated by adding 15 mM iodoacetamide for 1 hour at 25°C in the dark. The excess of iodoacetamide was quenched by 2.5 mM dithiothreitol. Protein concentration was determined by BCA assay using bovine serum albumin as standard. Proteins were digested at 37°C for 3 hours with Lys-C endoproteinase in a 1:100 (w/w) enzyme:substrate ratio. Then, 450μL of 50 mM ammonium bicarbonate were added to dilute urea. Samples were further incubated overnight at 37°C in the presence of modified porcine trypsin Gold in a 1:50 (w/w) enzyme:substrate ratio. The digestion was stopped by addition of 0.1% formic acid (FA) and peptide mixtures were centrifuged for 30 min at maximum speed (21,500 x g) at 4°C. Tryptic peptides present in supernatants were then subjected to desalting using reversed phase C18 spin columns as recommended by the supplier.

Peptide mixtures were prepared in 20 μL of 3% acetonitrile containing 0.1% FA (Solvent A) and analyzed on a Q-Exactive Plus (Thermo Fisher Scientific, San José, CA, USA) coupled to a Proxeon Easy nLC 1000 reversed phase chromatography system (Thermo Fisher Scientific, San José, CA, USA) using a binary solvent system consisting of solvent A and solvent B (0.1% FA in acetonitrile). 500 ng of tryptic digests were loaded on an Acclaim Pepmap C18 precolumn (2 cm x 75 μm i.d., 2 μm, 100 Å) equilibrated in solvent A and peptides were separated on an Acclaim Pepmap C18 analytical column (25 cm x 75 μm i.d., 2 μm, 100 Å) at a constant flow rate of 300 nL/min by two successive linear gradients of solvent B from 0% to 20% in 68 min, from 20% to 32% in 22 min and then up to 85% in 5 min followed by an isocratic step at 85% for 10 min. The instrument was operated in positive and data-dependent acquisition modes with survey scans acquired at a resolution of 70,000 (at m/z 200 Da) with a mass range of m/z 400–1,800. After each full-scan MS, up to 10 of the most intense precursor ions (except +1 or unassigned charge state ions) were fragmented in the HCD cell (normalized collision energy fixed at 27) and then dynamically excluded for 60 s. AGC target was fixed to 3x10^6^ ions in MS and 10^5^ ions in MS/MS with a maximum ion accumulation time set to 100 ms for MS and MS/MS acquisitions. All other parameters were set as follows: capillary temperature, 250°C; S-lens RF level, 60; isolation window, 2 Da. Acquisitions were performed with Excalibur software (Thermo Fisher Scientific, San José, CA, USA) and to improve mass accuracy of full-scan MS spectra, a permanent recalibration of the instrument was allowed using polycyclodimethylsiloxane ((C_2_H_6_SiO)_6_, m/z 445.12003 Da) as lock mass.

### Processing of proteomic data

Raw Orbitrap data were processed with Proteome Discoverer 2.1 software (Thermo Fisher Scientific, San José, CA, USA) and searched against the UniProtKB database restricted to the *C*. *reinhardtii* taxon (15,175 entries on 2017.05.12) using an in-house Mascot search server (Matrix Science, London, UK; version 2.4). Mass tolerance was set to 10 ppm for the parent ion mass and 20 mmu for fragments and up to two missed cleavages per peptide were allowed. Methionine oxidation, N-terminal acetylation of peptides and deamidation of asparagine and glutamine were taken into account as variable modifications and cysteine carbamidomethylation as fixed modification. Peptide False Discovery Rates (FDRs) were determined by searching against a reversed decoy database and peptide identifications were filtered at 1% FDR using the Percolator node. Proteins were validated if they were identified with at least two different peptides passing the peptide FDR filter. For each type of preparation (MX-CW15; SS-AT; WT-cell, WT-cp) two biological replicates and two analytical replicates were analyzed. In Proteome Discoverer 2.1 software, peptide abundance values were quantified according to areas from their eXtracted Ion Chromatograms (XICs) using the Precursor Ions Area Detector and Peptide Quantifier nodes. For each technical replicate, protein abundance values were calculated by summing abundance values of all unique and razor peptides available using the Protein Quantifier node and, for each biological replicate, protein abundance values consisted of the average of two technical replicates. As similar amounts of tryptic digest (500 ng) from preparations supposed to be highly different (MX-CW15; SS-AT; WT-cell, WT-cp) have been injected for LC-MS/MS analyses, no normalization was performed before comparisons.

The mass spectrometry proteomics data are summarized in S1 Table and have been deposited to the ProteomeXchange Consortium via the PRIDE partner repository with the dataset identifier PXD004509 (http://proteomecentral.proteomexchange.org; Reviewer account details: Username: reviewer15271@ebi.ac.uk; Password: 9fcT5WTO).

## Results

### Analyses of pyrenoid-enriched fractions

**Fig 2:**
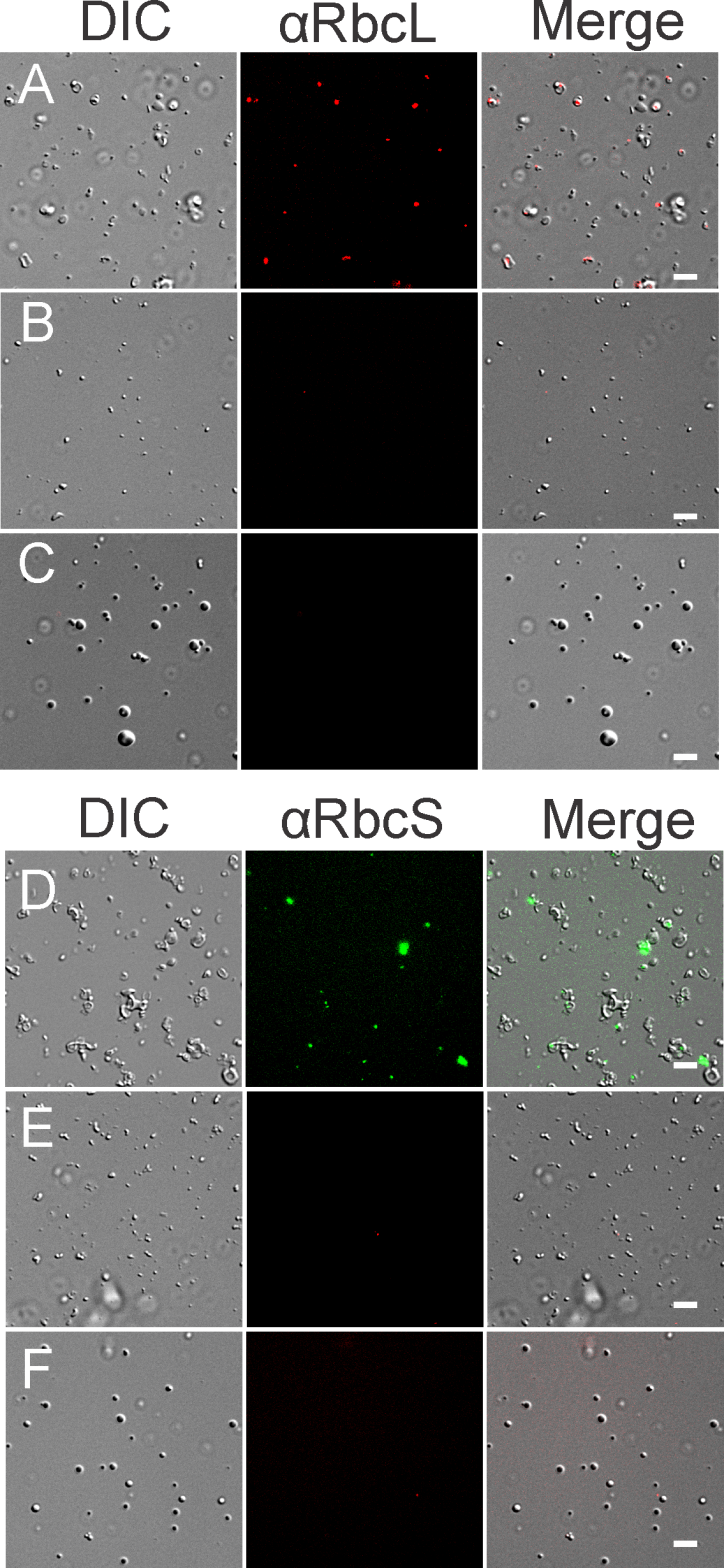
Pyrenoid-enriched fractions contain pyrenoids as revealed by IF microscopy. P fractions were shown to contain pyrenoids, seen as spherical bodies of *ca*. 1 μm that IF stain for marker proteins for the pyrenoid; RbcL and RbcS. Columns show images from differential interference contrast (DIC) microscopy, IF staining for RbcL (A-C) or RbcS (D-F), and the merged images for pyrenoid-enriched fractions from the WT strain (A and D), and the pyrenoid-deficient strains: *ΔrbcL* (B and E) and *SSAT* (C and F). Size bars = 10 μm.

We first developed further a protocol for the preparation of pyrenoid-enriched pellet (P) fraction as outlined in Fig 1A and Methods [26, 27]. With P fractions prepared from cells of the wild-type (WT) strain, MS analysis revealed the presence of many protein contaminants from diverse intracellular compartments, *e*.*g*. mitochondria, cytoplasm, and ER (S1 Fig). To minimize these contaminants, we purified chloroplasts and prepared P fractions from them. Immunoblot analyses revealed that the isolated chloroplasts were, relative to total cellular protein content, depleted at least 7-fold of endoplasmic reticulum (BIP), 15-fold of mitochondria (AOX1), and 500-fold of cytoplasm (CyL4) (Fig 1B). Most chloroplasts were retained during isolation, as revealed by the retention of the chloroplast ribosomal protein L7/L12 and the large subunit of Rubisco (RbcL) (Fig 1B). From these chloroplasts, P fractions were prepared and subjected to analysis by SDS-PAGE and silver-staining (Fig 1C). P fractions prepared from isolated chloroplasts versus cells showed distinct protein compositions with several bands specifically enriched in one or the other (Fig 1C). All pyrenoid-enriched fractions were highly enriched for RbcL and the small subunit of Rubisco (RbcS), both serving as markers for pyrenoids. Using fluorescence microscopy, we verified that P fractions indeed contain pyrenoids, which were seen as spherical bodies that were immunofluorescence (IF)-stained for RbcL and RbcS and had the expected diameter of 1-2 μm (Fig 2A and 2D). As expected, P fractions obtained from the pyrenoid-deficient mutants lacked spheres with RbcL or RbcS (Fig 2B, 2C, 2E and 2F). Finally, results of MS analysis of the P fractions from isolated chloroplasts revealed primarily proteins that are known or predicted to be in the chloroplast (S1 Fig). This represents a drastic improvement over P fractions from cells. In summary, we have improved existing protocols for the purification of chloroplasts and pyrenoids. We overcame the problem of pyrenoid instability and eliminated the need for mercury chloride [26, 27]. Nevertheless, our P fractions contained non-pyrenoid material, seen as structures that did not IF-stain for RbcL or RbcS (Fig 2). Therefore, it was necessary to use additional criteria to specifically identify pyrenoid proteins in P fractions.

### Analyses of pyrenoid-deficient mutants identified contaminant proteins

It is known that many proteins in the chloroplast of vascular plants (which lack a pyrenoid) become insoluble in the presence of Triton X-100 due to their particular physicochemical properties or being in a supramolecular assembly [33]. To identify such contaminants in P fractions from WT, P fractions from two mutants that lack a pyrenoid were prepared and analyzed by MS in parallel (Table 1). These control P fractions should have only contaminant proteins, for subtraction from the proteome of P fractions that contained pyrenoids. The *ΔrbcL* mutant carries a disruption of the chloroplast gene encoding RbcL and lacks a pyrenoid [24, 34]. Analysis by SDS-PAGE and silver staining revealed that P fractions from *ΔrbcL* lacked both RbcL and RbcS, which is known to have a very low abundance in RbcL-deficient mutants [25] (Fig 1D). The Rubisco deficiency of *ΔrbcL* considerably alters cellular metabolism and might therefore alter the non-pyrenoid proteins in the P fraction, thereby biasing the subtraction of contaminants. To circumvent this potential drawback, we also used *SSAT*, a strain that has Rubisco (and is photoautotrophic) but does not assemble a pyrenoid (see Materials and Method) [22]. Therefore, it constitutes an appropriate control strain for the subtractive proteomic strategy. Results of SDS-PAGE and silver staining revealed a few proteins in common between P fractions from the pyrenoid-deficient and WT strains (indicated by asterisks in Fig 1D, providing confidence that results from these control strains, indeed, identify contaminant proteins for subtraction and refinement of the pyrenoid proteome. P fractions from SSAT lacked RbcS (Fig 1D) but occasionally contained a small amount of RbcL, which was possibly of a non-Rubisco RbcL pool that is insoluble in Triton X-100 [25]. Nevertheless, by using a quantitative mass spectrometry approach we were able to include in the pyrenoid proteome RbcL and many other proteins detected at low levels in the control P fractions based on the relative abundance of these proteins in each sample as detailed in the following sections.

**Table 1:** Strains used in this study.

Name	Genotype	Pyrenoid	CCM	Rubisco	RbcL	RbcS	reference
*KA6*	*CW15*	+	+	*+*	+	+	This study
*ΔrbcL* *(MX3312)*	*ΔrbcL; CW15*	-	-	*-*	-	trace	[24, 25]
*SSAT*	*ΔrbcS1; ΔrbcS2; CW15*, transgenic for the *RBCS1B gene* of *A*. *thaliana*	-	impaired	+	+	+	[23]

### Mass spectrometry analysis

To characterize the pyrenoid proteome, proteomic analyses were performed using accurate mass/high resolution tandem mass spectrometry on P fractions prepared from WT cells (WT-cell) or from chloroplasts isolated from WT cells (WT-cp) or isolated from cells of the pyrenoid-deficient strains; *ΔrbcL* (MX-CW15) and *SSAT* (SS-AT) (Table 1). For each sample, two independent biological replicates, and two technical replicates of each, were analyzed by nLC-MS/MS. For the processing of mass spectrometry data, proteins were not only identified using the MASCOT software with stringent validation criteria (2 different peptides having a peptide False Discovery Rate < 0.01) but were also quantified by summing the intensities of precursor ions that correspond to unique peptides belonging to proteins. Such a XIC-based relative quantification strategy allowed us to compare abundance values of proteins present in the different P fractions and determine proteins that are specific to the pyrenoid from those that can be considered as contaminants. It should be mentioned that Proteome Discoverer 2.1 does not use systematically the same set of peptides to calculate protein abundance across different samples. For this reason, protein abundance values generated by this software should be considered as semi-quantitative values rather than quantitative ones.

We first calculated for each type of preparation (WT-cp, WT-cell, MX-CW15 and SS-AT) a significance threshold above which proteins could be considered as significantly enriched. This was done by calculating logarithmized abundance ratios for all proteins present in the two biological replicates and by calculating the corresponding significance threshold as follows:

Significance Threshold = Global Mean Ratio + 2 x Standard Deviation (S1 Table).

The number of proteins showing ratios higher than these thresholds could be estimated to less than 3.9% for each type of preparation. This constitutes a rough estimation of false positives in our conditions. These calculated thresholds were then used to compare protein abundance in P fractions prepared from different sources (WT, *ΔrbcL* and *SSAT* strains). When comparing two samples with distinct thresholds, only the higher significance threshold was taken into account (S2 Table). Using this stringent criterion that considerably decreased false positives, we quantitatively compared proteins present in P fractions prepared from chloroplasts isolated from WT, *ΔrbcL* and *SSAT* strains. Therefore, proteins were considered to belong to the pyrenoid if their abundance ratio was found to be higher than 9.41 and 7.04 when comparing WT with MX-CW15 and SS-AT samples, respectively (S2 Table).

**Fig 3:**
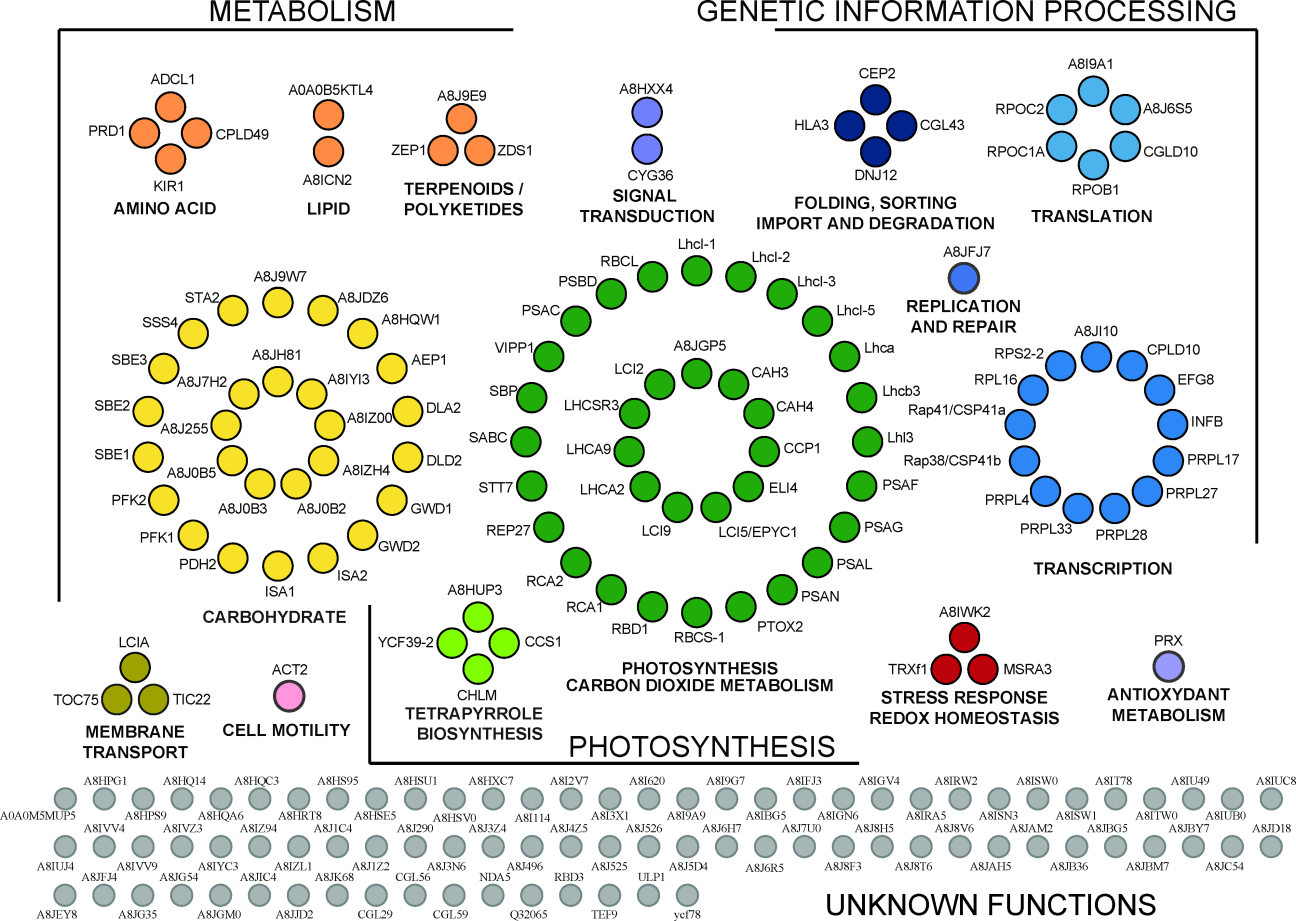
Functional annotation of the 190 proteins of the pyrenoid proteome. Proteins were classified according to KEGG for *C*. *reinhardtii* using a three Gene Ontology annotation levels. The KEGG annotations were further refined manually to optimize functional categories. Proteins involved in CO_2_ fixation, CO_2_ metabolism (such as carbonic anhydrases) or annotated as `low CO_2_ inducible`genes are included in node “Photosynthesis and CO_2_ metabolism”. The node “Carbohydrate” has proteins in carbohydrate (principally starch) metabolism and proteins harboring an alpha amylase catalytic domain or a starch-binding domain. Nodes are labeled either with the gene name or with the UniprotKB identifier. The functional categories and the properties of each protein are detailed in S3 Table.

Comparison of these samples revealed that 105 proteins were present in P fraction prepared from chloroplasts isolated from WT strain and absent in the control samples. In addition, our quantitative proteomic analysis revealed that 85 proteins were strongly enriched in P fractions from WT compared to P fractions from *ΔrbcL* and *SSAT* strains. This underlines the advantage of such a quantitative approach as these proteins would have been considered as contaminants with a simple binary approach, *i*.*e*. one based on the presence or absence of the proteins. For example, two of the most abundant proteins of the pyrenoid, RbcL and Rubisco activase (RCA1) belong to this list of 85 proteins and would have been missed without quantitative analysis. Indeed, the proteins identified in each sample were highly abundant in pyrenoid enriched fractions from WT chloroplasts while they were present as traces in samples from control strains (S1 Table). The resulting pyrenoid proteome contains 190 proteins involved in diverse cellular pathways and processes (Fig 3, S3 Table).

### Functional analysis of the pyrenoid proteome

The 190 proteins identified were classified according to their subcellular localization (S2 Fig). The intracellular localization of each protein (except those encoded by the chloroplast genome and, therefore, known to be in this organelle) was predicted using Predalgo, a program that predicts the intracellular localization of a query protein in green algae to one of three intracellular compartments: the chloroplast, mitochondria, or the secretory pathway [35]. A majority (60%) of the proteins are predicted to be located in the chloroplast compartment. This is in sharp contrast with the total theoretical proteome that only contains 20% of proteins predicted to be localized to the chloroplast (S2 Fig). Nevertheless, more than one third of the proteins are predicted to be localized outside of chloroplasts even though the samples were prepared from purified chloroplasts. This most likely reflects limitations in the discrimination capacity of the Predalgo algorithm. Consistently, the proteome of the chloroplast stroma in *C*. *reinhardtii*, which contains numerous proteins used to train Predalgo, contains 29% non-chloroplast proteins. Altogether, the subcellular localization of most of the proteins identified using our subtractive proteomic strategy is consistent with the localization of the pyrenoid microcompartment within the chloroplast [2, 36]. The 190 proteins of the pyrenoid proteome were classified according to the Kyoto Encyclopedia of Genes and Genomes (KEGG) for *C*. *reinhardtii* and using a limited number of Gene Ontology annotation levels (Fig 3) [37]. The results support the roles of the pyrenoid as a hub of carbon metabolism and in roles relating to the plastid genetic system as detailed in the Discussion section.

### Evidence for localized synthesis of RbcL at the pyrenoid by plastid ribosomes

The presence of many proteins of the chloroplastic translation machinery suggests that protein synthesis occurs in physical association with the pyrenoid (Fig 3, S3 Table). If these ribosomes are active in translation, previous results suggest two possibilities. First, these ribosomes could be bound to the “chloroplast translation membranes” that were detected adjacent to the pyrenoid, possibly physically connected to it, thereby resulting in their co-purification with pyrenoids. Because these membranes are specialized in the synthesis and assembly of PSII subunits encoded by the plastid genome [38], this hypothesis predicts that newly synthesized PSII subunits would be detected in the P fraction of a WT strain. Second, these ribosomes could be translating the *rbcL* mRNA at the outer surface of the pyrenoid [39]. This predicts that P fractions contain newly synthesized RbcL.

**Fig 4:**
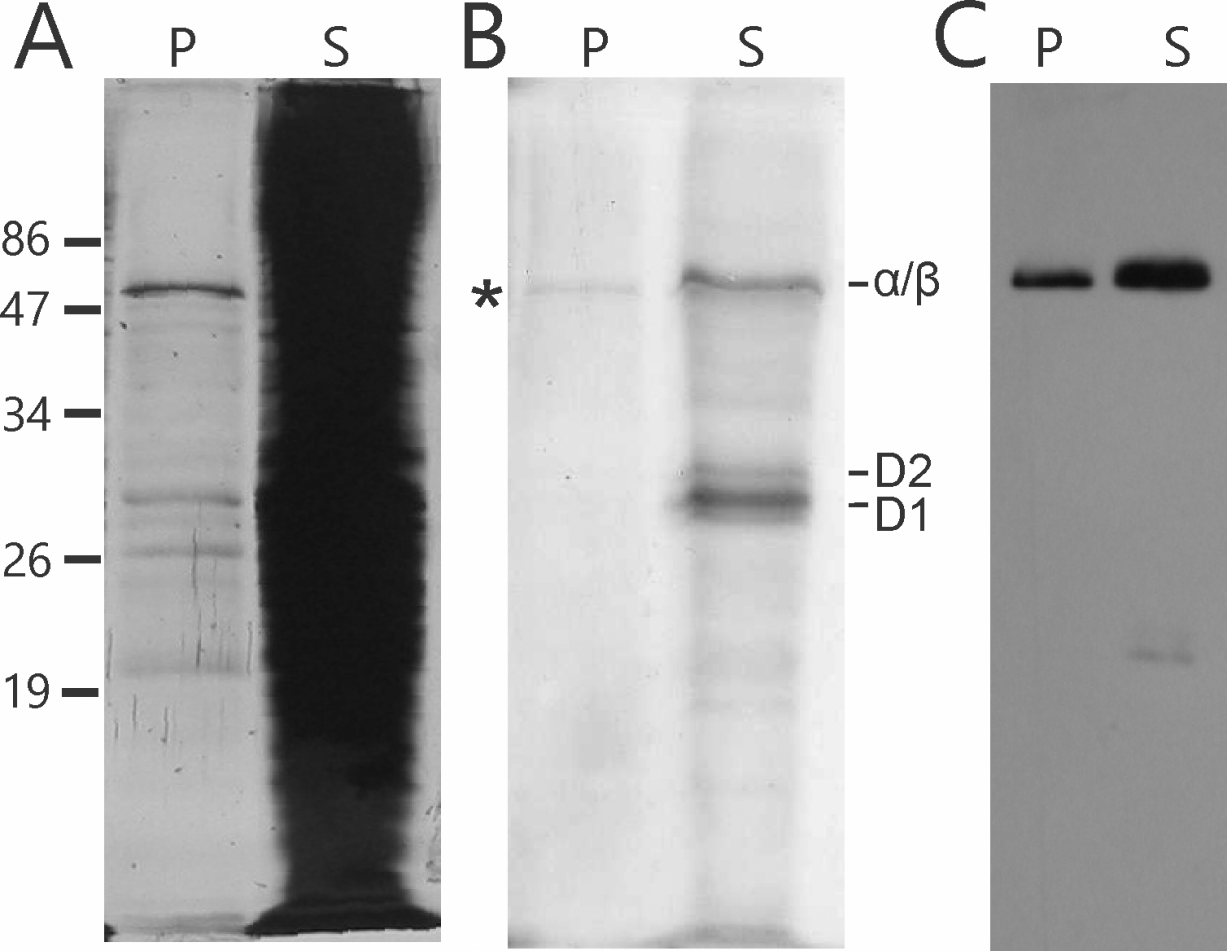
Newly synthesized RbcL was detected in the pyrenoid fractions. Proteins in the pyrenoid-enriched P and supernatant S fractions were resolved by SDS-PAGE and analyzed by (A) silver-staining, (B) phosphorimaging of ^35^S-labeled proteins during a 5 min pulse and (C) immunoblot analysis for RbcL. A newly synthesized ^35^S-pulse-labeled protein with the molecular mass of RbcL (55 kDa) was detected in the pyrenoid-enriched pellet (P) fraction (RbcL). ^35^S-pulse-labeled thylakoid membrane proteins are D1, D2 and the co-migrating α and β subunits of the ATP synthase of the chloroplast.

To distinguish between these two possibilities, we asked whether the pyrenoid-enriched fractions have newly synthesized proteins and, if so, whether they are PSII subunits, RbcL, or both. Short (5 min) ^35^S-pulse radiolabeling experiments were carried out with intact WT cells. To radiolabel only proteins synthesized by the bacterial-like 70S ribosomes in the chloroplast, cycloheximide was used to inhibit translation by 80S cytoplasmic ribosomes [40]. These cells were then fractionated into S and P fractions and, after separation by SDS-PAGE [^35^S] proteins were detected by phosphorimaging. Most newly synthesized (^35^S-labelled) proteins were visualized in the supernatant fraction, including the PSII subunits D1 and D2 (Fig 4B). However, in the P fraction, we detected only one radiolabeled protein, with the molecular mass of RbcL (55 kDa). This probably corresponds to newly synthesized RbcL that pelleted due to close association with pyrenoids. These results demonstrate that the chloroplast ribosomes did not pellet with pyrenoids due to an association with chloroplast translation membranes and they support the pyrenoid being the location of *rbcL* mRNA translation [39].

## Discussion

Our results support the view of the pyrenoid as a hub for the metabolism of CO_2_ and starch. The pyrenoid proteome reported here provides directions for the exploration of pyrenoid functions, the CCM of green algae, and will serve as a resource for use of pyrenoids to enhance agricultural plant productivity. Below, we address a few issues and caveats. Then we discuss how our results advance our understanding of the functions of the pyrenoid.

Subtractive proteomic approaches can provide stringency in the characterization of an intracellular compartment if it is possible to partially purify that compartment and obtain cells lacking it. The quality of such subtractive proteomic analyses is strongly enhanced if mass spectrometry analyses are conducted at least in a semi-quantitative way. Our analyses of control P fractions from pyrenoid-deficient mutants, along with an improved chloroplast isolation procedure reported here (Materials and Methods), eliminated many contaminating proteins from our pyrenoid proteome. However, a few concerns remained. Because pyrenoid tubules are contiguous with thylakoid membranes and contain chloroplast stroma [8], we considered the possibility that they contributed thylakoid and stromal proteins to the pyrenoid proteome. However, this was not a major problem because our pyrenoid proteome shows a strong bias against proteins from these compartments. Of the proteins of thylakoid membranes, we found primarily protein subunits of PSI and its light-harvesting complex. There is only one subunit of PSII, and no subunits of the two remaining major complexes; the cytochrome b6/f complex, and ATP synthase (Fig 3, S3 Table). Similarly, our pyrenoid proteome does not resemble the proteome of the chloroplast stroma in *C*. *reinhardtii*; it contains only 14 proteins of the 274 reported in the stroma proteome (S3 Fig) [2].

Two caveats should be considered. First, our results do not demonstrate that any of the 190 proteins in Fig 3 and S3 Table are localized primarily to the pyrenoid. Second, our proteome is incomplete because MS analyses are not exhaustive since they do not detect peptides from all proteins in a sample. Moreover, proteins of the pyrenoid-deficient control strains that are localized to the pyrenoid in WT cells could have incidentally fractionated to the control P fractions by virtue of their physicochemical properties or presence in supramolecular assemblies and therefore may have been subtracted inappropriately from the proteome.

### Our proteome substantiates the role of the pyrenoid in CO_2_ metabolism

The pyrenoid in *C*. *reinhardtii* contains four major proteins, which are involved in CO_2_ fixation and of comparable abundance: RbcL, RbcS, RCA1 and EPYC1 (LCI5[10, 18]**E**). Along with these proteins, we identified a total of 11 proteins with known or suspected functions in CO_2_ metabolism (S3 Table). These proteins are required for Rubisco activity/assembly or for CCM. Of these, 6 are regulated by low [CO_2_] (S3 Table) including Low-CO_2_-inducible protein 5 (LCI5), Low-CO_2_-inducible chloroplast envelope protein (CCP1), carbonic anhydrase 3 (CAH3), and mitochondrial carbonic anhydrase beta type (CAH4). CAH3 generates CO_2_ from the bicarbonate that is imported into the pyrenoid from the chloroplast stroma [41] and it is localized to the pyrenoid [42]. While CAH4 accumulates under low-CO_2_ conditions in mitochondria [43], our results suggest that it is also localized to the chloroplast.

### Our proteome supports the role of the pyrenoid in starch metabolism

We identified 27 proteins in the KEGG 2 category “Carbohydrate Metabolism” (Fig 3, S3 Table). Of these, 20 proteins are in the KEGG3 category “Starch and Sucrose Metabolism”, although most (19) have starch metabolic functions that are either known or predicted. Five proteins function in starch biosynthesis: two starch synthases (STA2, SSS4) and three starch branching enzymes (SBE1-3). Five proteins function in starch degradation: an alpha-amylase, two isoamylases (ISA1-2) and two alpha-glucan water dikinases (GWD1-2) [12]. In addition, nine proteins have predicted starch-related functions based on their sequences which contain an alpha amylase catalytic domain or a starch-binding domain.

These results provide the first direct evidence that starch biosynthesis and degradation occur in the pyrenoid, a long-standing hypothesis [12]. The pyrenoid was proposed to be a location of starch biosynthesis because it is surrounded by starch plates whose biogenesis coincides with the induction of CCM by the pyrenoid under low CO_2_ conditions [44]. However, this hypothesis has been challenged based on the ability of pyrenoid-deficient mutants to produce the starch in granules located in the chloroplast stroma, away from the pyrenoid [12]. These results are reconciled if the pyrenoid metabolizes the starch that surrounds it, but not the starch of the chloroplast stroma.

### Photosynthesis

The pyrenoid proteome has subunits of PSI and its light-harvesting complex (Fig 3, S3 Table). These are consistent with the previous histochemical staining of PSI in pyrenoid tubules [10]. The one subunit of PSII identified is probably a contaminant because none of the 24 other PSII subunits were identified or the highly abundant proteins of the light-harvesting complex of PSII. Moreover, previous results based on histochemical staining and electron microscopy suggest pyrenoid tubules in red algal species lack PSII [10].

Six proteins of the pyrenoid proteome have known or proposed functions in responses to light. These include the STN7/Stt7, the protein kinase that regulates “state transition” responses to variable light quality and an early light-induced protein (ELIP), and a stress-related light-harvesting proteins (LHCSR3). Finally, the proteome has zeaxanthin epoxidase; an enzyme of the xanthophyll cycle which functions in non-photochemical quenching [45]. Thus, the pyrenoid might have a role in photoacclimation, responses to light-induced stress, or both.

### Metabolism

Of the 109 proteins with annotated functions, 74 (68%) are in the KEGG1 category “Metabolism” (Fig 3, S3 Table). Besides photosynthesis and starch metabolism that constitute the two main categories, we detected proteins involved in pigment metabolism. Four proteins function in the metabolism of porphyrin and chlorophyll: pyridine nucleotide binding protein YCF39, Mg-protoporphyrin-IX S-adenosyl methionine O-methyl transferase CHLM, Cytochrome c biogenesis protein CCS1 and a phycocyanobilin ferredoxin oxidoreductase-like protein. Three proteins participate in carotenoid biosynthesis: zeta-carotene desaturase ZDS1, carotenoid isomerase and zeaxanthine epoxidase ZEP1. Also in metabolism are proteins that function in amino acid metabolism (3), in C metabolism (7) (excluding starch and sucrose metabolism), in S metabolism (2), and in lipid metabolism (2). Thus, in addition to the metabolism of energy (photosynthesis), starch and CO_2_, our results further support the long-standing view of the pyrenoid as a compartment for intermediary metabolism.

### Translation and RNA metabolism

Our pyrenoid proteome contains 13 proteins of the translation machinery of the chloroplast. Of these, 11 are proteins in the proteome from the 70S ribosome of the chloroplast (Fig 3, S3 Table) [46, 47]. These include two proteins that are associated with the 70S chloroplast ribosome; Rap41 and Rap38 [47]. These are homologues of NAD-dependent epimerase/dehydratases, which bind RNA and have endoribonuclease activity *in vitro*, called in vascular plants CPS41a and CPS41b, respectively [48, 49]. Genetic evidence in *A*. *thaliana* supports roles of CPS41a and CPS41b in transcription and translation [50]. Evidence exists for protein synthesis in association with the pyrenoid in *C*. *reinhardtii*. *In situ* localization revealed newly synthesized proteins in pyrenoid tubules by radiolabeling and EM autoradiography [51]. Results of IF confocal microscopy revealed chloroplast ribosomes both within and adjacent to the pyrenoid, regions called “chloroplast stress granules” and the “translation zone” respectively [15, 52]. Chloroplast stress granules receive mRNAs from disassembled polysomes and return them to the translated pool, suggesting that these structures and the pyrenoid have a role in spatially organizing the initiation of translation in the chloroplast [15]. In addition, the surface of the pyrenoid could serve as a platform for translation because the *rbcL* mRNA was seen to localize at the pyrenoid periphery in a translation-dependent manner [39] and we detected a newly synthesized protein, most probably RbcL, in P fractions, supporting the pyrenoid as a location of RbcL synthesis (Fig 4).

Our pyrenoid proteome also contains enzymes related to RNA metabolism: polyribonucleotide phosphorylase/nucleotidyltransferase, a DEAD box RNA helicase and several subunits of the plastid-encoded RNA polymerase of the chloroplast (Fig 3, S3 Table). The putative polyribonucleotide phosphorylase/nucleotidyltransferase supports the previously proposed role of the pyrenoid in oxidized RNA quality control in the chloroplast [25] because, in both bacteria and mammals, this protein binds oxidized RNA and is required for oxidative stress tolerance [53–55].

### Proteins of unknown function

We also identified 81 proteins of unknown function. These proteins are candidates for new proteins that function in processes that occur in pyrenoids. Comparison of these proteins with low CO_2_ inducible genes identified by RNA-seq revealed 22 proteins that are likely involved in CCM or CO_2_ metabolism within pyrenoids (Fig 3, S3 Table) [56].

### Similarities of the pyrenoid and carboxysome proteomes

Comparison of the proteomes of the pyrenoid and the functionally analogous compartment in cyanobacteria, the carboxysome, revealed similarities that were expected and unexpected. Carboxysomes are the major location of CO_2_ fixation by Rubisco and they have a CCM [11]. The proteome of a carboxysome-enriched fraction from *Synechococcus* PCC7942 has, as expected, both subunits of the Rubisco holoenzyme and CCM proteins. Like the pyrenoid proteome, the carboxysome proteome has no additional Calvin-Benson cycle enzymes. Unexpected proteins with functional similarities were found in the proteomes of both the pyrenoid and carboxysomes: nucleic acid-binding proteins, a predicted helicase, a DNAJ chaperone, RNA polymerase subunits, and ribosomal proteins [57]. Together, these results suggest that carboxysomes and pyrenoids have analogous functions relating to gene expression, in addition to their known common role in CO_2_ fixation.

## Supporting information

S1 Table Significance threshold determination between biological replicates of the four sample types (MX-CW15, SS-AT, WT-cp, WT-cell). (XLSX)

S2 Table Significance thresholds used for pairwise comparison of protein abundance in P fractions prepared from the different samples (MX-CW15, SS-AT, WT-cp, WT-cell). (XLSX)

S3 Table Properties of the 190 proteins present in the *Chlamydomonas* pyrenoid proteome. (XLSX)

S1 Fig Distribution of the subcellular localization of proteins identified in P fractions prepared from WT cells (WT-cell) or from WT purified chloroplasts (WT-cp). Pie charts represent the breakdown by intracellular compartment for proteins identified in WT-cell (A) and WT-cp (B) samples (S1 Table). Chloroplast and mitochondrial encoded proteins were placed in the corresponding subcellular fractions. Localization of proteins encoded by the nuclear genome was predicted using the Predalgo prediction program [35].
(PDF)

S2 Fig Distribution of the subcellular localization of proteins present in diverse proteomes. Pie charts represent the breakdown of proteins by intracellular compartment for (A) the pyrenoid proteome (this study, S3 Table), (B) the stromal proteome [2], (C) the chloroplast proteome [36] and (D) the total proteome (derived from the genome sequences). Chloroplast and mitochondrial encoded proteins were placed in the corresponding subcellular fractions. Localization of proteins encoded by the nuclear genome was predicted using the Predalgo prediction program [35].
(PDF)

S3 Fig Overlap between the pyrenoid proteome and the stromal proteome. Venn diagram showing the limited overlap between the pyrenoid proteome (present study) and the stromal proteome [2]. The 14 proteins common to both proteins are listed in S3 Table.
(PDF)
