# Supplementary material for: Pyrenoid functions revealed by proteomics in Chlamydomonas reinhardtii
Source: PLoS One. 2018 Feb 26;13(2):e0185039. doi: 10.1371/journal.pone.0185039 (PMC5826530; doi:10.1371/journal.pone.0185039)
Supplement: S1 Fig — Pie charts represent the breakdown by intracellular compartment for proteins identified in WT-cell (A) and WT-cp (B) samples (S1 Table). Chloroplast and mitochondrial encoded proteins were placed in the corresponding subcellular fractions. Localization of proteins encoded by the nuclear genome was predicted using the Predalgo prediction program [35]. (PDF) [file pone.0185039.s004.pdf]

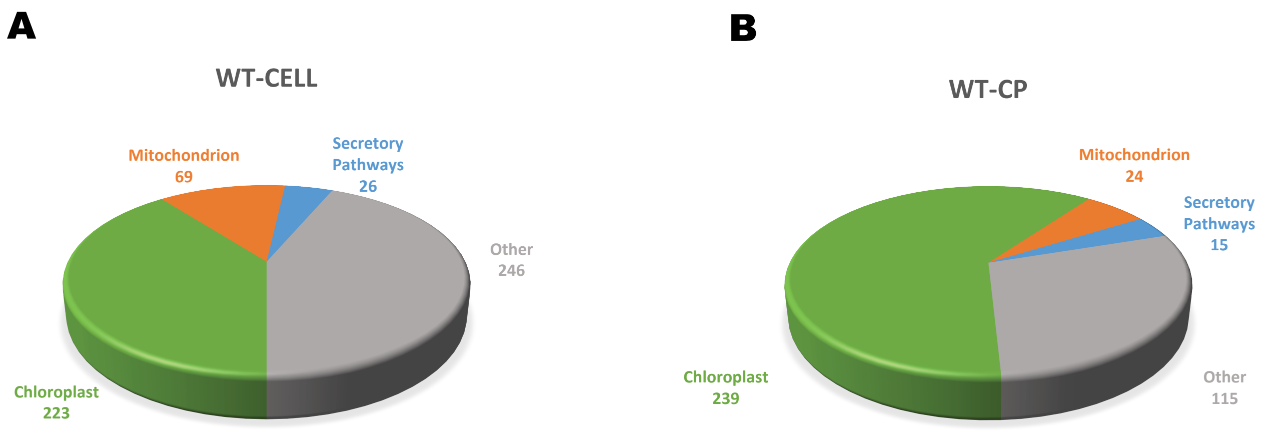

S1 Fig.
Distribution of the subcellular localization of proteins identified in P fractions prepared from WT cells (WT-cell) or from WT purified chloroplasts (WT-cp).
