# Supplementary figures and images for: Pyrenoid functions revealed by proteomics in Chlamydomonas reinhardtii
Source: PLoS One. 2018 Feb 26;13(2):e0185039. doi: 10.1371/journal.pone.0185039 (PMC5826530; doi:10.1371/journal.pone.0185039)

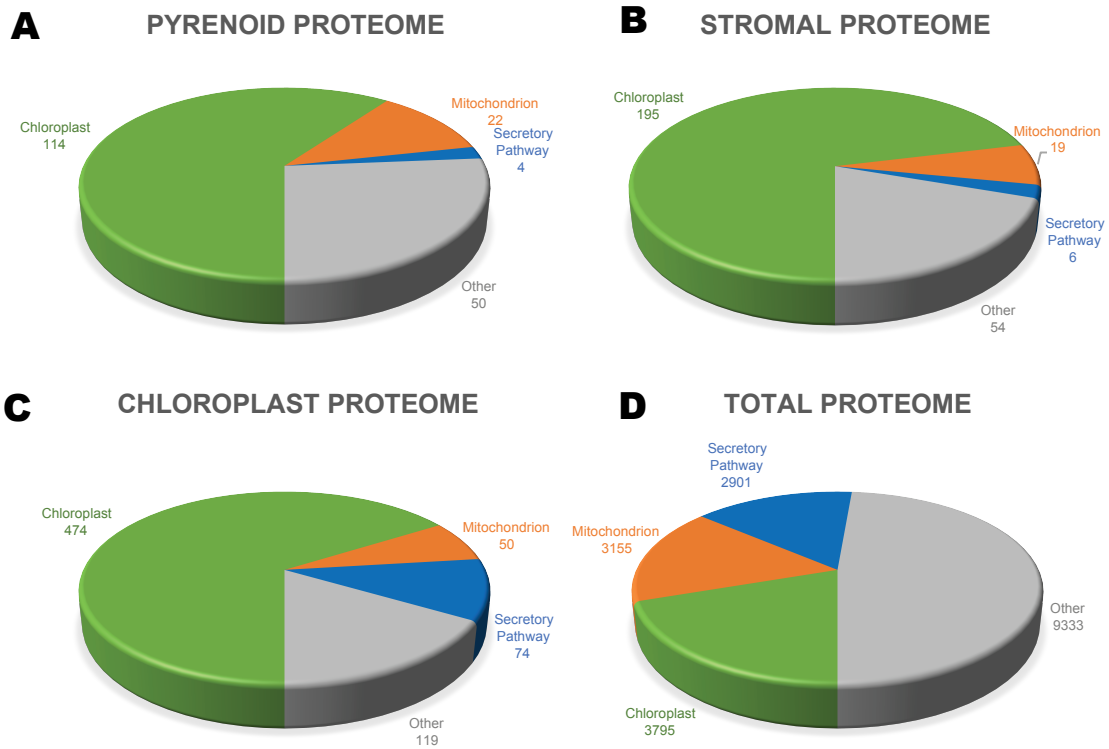

**S2 Fig.**  
**Distribution of the subcellular localization of proteins present in diverse proteomes.**

Supplement: S2 Fig — Pie charts represent the breakdown of proteins by intracellular compartment for (A) the pyrenoid proteome (this study, S3 Table), (B) the stromal proteome [2], (C) the chloroplast proteome [36] and (D) the total proteome (derived from the genome sequences). Chloroplast and mitochondrial encoded proteins were placed in the corresponding subcellular fractions. Localization of proteins encoded by the nuclear genome was predicted using the Predalgo prediction program [35]. (PDF) [file pone.0185039.s005.pdf]

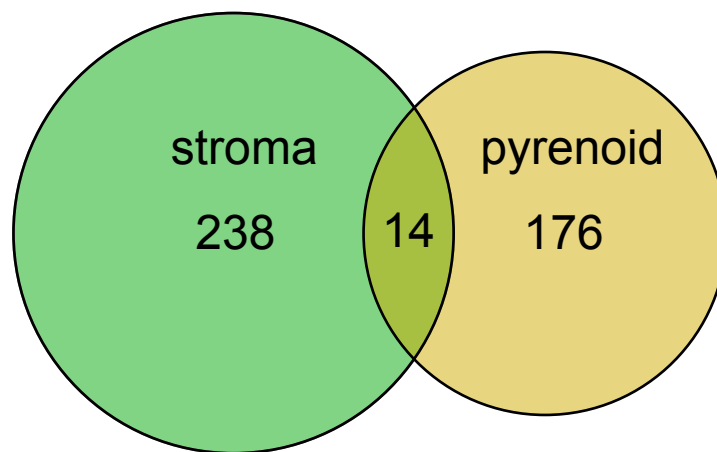

**S3 Fig.**  
**Overlap between the pyrenoid proteome and the stromal proteome**

Supplement: S3 Fig — Venn diagram showing the limited overlap between the pyrenoid proteome (present study) and the stromal proteome [2]. The 14 proteins common to both proteins are listed in S3 Table. (PDF) [file pone.0185039.s006.pdf]
